# Models connecting microstructure and charge transport in disordered semiconducting polymers: from theories to digital design

**DOI:** 10.1039/d5mh01079a

**Published:** 2025-08-09

**Authors:** Colm Burke, Alessandro Troisi

**Affiliations:** a Department of Chemistry, University of Liverpool Liverpool L69 3BX UK a.troisi@liverpool.ac.uk

## Abstract

Theoretical methods connecting chemical composition, structure, electronic properties and charge transport properties are reviewed with focus on the approaches used to connect between models at different resolutions. Within each scale of modelling the research field has reached a good level of maturity and consensus between practitioners. Phenomenological models can now be fully justified by microscopic models derived from first principles. The latter can be parametrized from atomistic models combining classical simulations of realistic systems, to obtain the microstructure, and electronic structure calculations. The throughput of such atomistic models has improved substantially and they can now be faster than synthesis and characterisation of novel polymers. Overall, the community has now achieved the capability of performing computer-aided design of semiconducting polymers with the expectation that the next generation of materials will be, for the first time, digitally designed.

## Abstract 2

Theoretical methods connecting chemical composition, structure, electronic properties and charge transport properties are reviewed with focus on the approaches used to connect between models at different resolutions.

## Graphical abstract

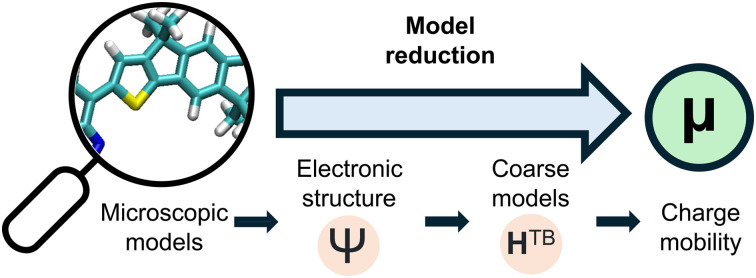

**Wider impact**

Unlike other areas of organic electronics, the approaches to model polymeric semiconductors are more rarely reviewed and the connections between different streams of research (phenomenological models, quantum dynamics propagation, atomistic simulations) are not immediately clear from the segregated literature. Designing polymers ultimately requires linking their chemical composition to device-scale physics and awareness of modelling challenges across these scales. Reduced models are emerging as the ideal link between phenomenological models (providing rigorous justification for them) and detailed atomistic models (because they can be systematically derived from them). As the number of materials that can be explored within a given study increases, it is now easier to derive statistically meaningful structure-property relationships or to explore hypothetical materials. This review offers a starting point to reflect on how the area of polymeric semiconductors can now start benefitting from the approaches of digital materials discovery.

## 1. Introduction

Conjugated semiconducting polymers (SCP) have huge potential for the development of low-cost, light-weight and flexible electronic devices and constitute arguably the largest portion of semiconducting organic materials, yet their complexity has hindered the systematic development of theoretical models when compared to crystalline or disordered molecular solids. For the latter two material classes, well-established methods exist to connect the local structure with charge transport properties and the key methodologies are often reviewed and benchmarked,^1,2^ with high-throughput virtual screening having been used to great effect in these areas.^3^

In contrast, attempts to do the same for polymers are more difficult and thus more scattered: polymer microstructure is complex and varied due to the huge chemical space available combined with the weakly-bonded macromolecular nature of chains inducing many conformational degrees of freedom.^4^ In addition, the polydispersity of the sample and processing techniques used in preparation contribute significantly to variations in measured experimental mobilities.^5^ The doping of polymer films,^6^ and distinct models that tackle the (comparatively rare but important) existence of crystalline phases,^7^ while not covered in this review, introduce yet more complications. It is therefore not an easy task to find a link between the chemical structure of constituent monomers, the microstructure, and the transport properties of a polymer – to say nothing of deriving generalisable design rules. It should be noted that, despite there being no consolidation into a standardised approach, improvements in state-of-the-art mobilities over the last several decades have been impressive,^4,8^ and design rules have been found. The paradigm of long-range order as a necessity for high-mobility was questioned when weakly ordered donor–acceptor copolymers emerged whose mobilities equalled or bettered their highly ordered predecessors,^9^ showing the continuing evolution of the field but also reaffirming that design rules may not be as steadfast as they seem, and that there may be multiple avenues to achieving exceptional device performance.

An important starting observation is that we are now able to construct fairly accurate microscopic models of amorphous polymers using a multitude of experimental and computational techniques. The more traditional crystallographic,^10,11^ optical,^12^ and nuclear magnetic resonance^13^ methods are now complemented by electron microscopy^14,15^ and scanning probe methods,^16^ allowing for a detailed picture of the microstructure to be built.^17^ Through advances in computing power and with more reliable force-field generation, classical molecular dynamics (MD) simulations are also consistently able to produce microscopic structural models that are congruent with experimental observables,^18–21^ and now at a much higher rate than previously possible.^22^ Emerging standards in experimental procedures, such as in the measurement of charge mobility in thin-film transistors,^23^ or the less controversial measure of space-charge limited current,^24^ give additional reason to believe that both the predictive and explanatory capabilities of charge transport models will improve.

Traditional theories of transport developed in the early days of organic electronics and based on variable-range hopping (VRH) have been very successful in accounting quantitatively for most observations at the device level.^25^ However, being phenomenological in nature, these theories did not allow for a connection to be made between microstructure and charge transport characteristics – a crucial component of systematic progress in materials development that becomes more and more important as the chemical space investigated experimentally widens and the possibility for similar mobilities to be achieved by chemically dissimilar polymers is increased. Several models based on a more detailed quantum mechanical propagation of the carrier wavefunction have been proposed but have been generally limited to simple benchmark systems^26,27^ and simplified Hamiltonians like polyacetylene^28^ or poly(*p*-phenylene vinylene) (PPV).^29^ In parallel, electronic structure calculations of realistic polymer systems have become readily available,^20,30^ suggesting that predictive and chemically detailed models are possible.

**Fig. 1:**
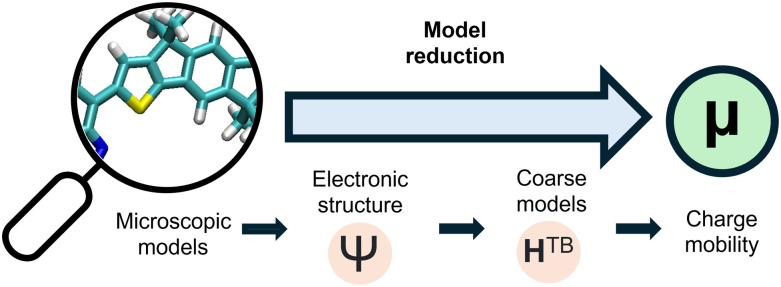
Schematic of the key ideas covered in this review.

As we discuss in the following sections, the derivation of a multiscale model connecting the local electronic structure of the polymer and the charge transport over tens of nanometres has proved to be a challenging task. The goal of this perspective is to provide an outline of the ongoing attempts to determine the relation between local microstructure and charge transport in disordered polymers (see Fig. 1). With this goal we assume that a valid microstructural model exists, and as such do not discuss the development of these models or the various effects that complicate their construction.^31^ We start by considering the three main branches of theoretical investigation: electronic structure calculations of detailed models in Section 2, kinetic or phenomenological coarse models in Section 3 and the study of simplified model systems in Section 4. We then look at the connection between scales either through fully atomistic models of transport (Section 5) and model reduction schemes (Section 6).

## 2. Electronic structure from polymer models

Computing the electronic structure of polymer models *via* quantum chemical methods is probably the most obvious way to form a direct link between chemical structure and electronic properties. Calculations performed on disordered SCPs cannot take advantage of periodicity in the same way as crystalline organic semiconductors: in disordered SCPs the problem is not reducible using symmetry. Compared to disordered molecular solids, where coupling between sites is considered isotropic and small, transport in disordered SCPs is anisotropic, with (relatively) fast hopping along the chain and much slower hopping between chains.^32–34^ Indeed, for systems with both crystalline and amorphous phases, one must resolve intra-chain, inter-chain, and inter-domain transport to have a comprehensive picture of the mobility of the material. For amorphous phases, or for polymers without semicrystalline domains, it is generally agreed however that the energy-dependent density of states (DOS) of a polymer – the availability of states to charge carriers – can be approximated as a superposition of the DOS of individual chain conformations.^35^ Since the DOS describes the energetic landscape of the polymer, various characteristics of its shape can be used as proxies for carrier mobility without requiring its explicit computation. The valence (conduction) band tail contains the states most relevant for hole (electron) transport, and as such its width or slope reflects the electronic disorder of the polymer.^36,37^ With disorder being the generally accepted cause of localisation in semiconducting polymers, as opposed to polaronic effects (static disorder in electronic coupling between monomers is much larger than the reorganisation energy),^38^ this descriptor is strongly correlated with the carrier mobility.^37^ As localisation of the wavefunction limits the distance charge carriers can ‘hop’ in variable-range hopping models, measures describing the extent of delocalisation of states relevant to charge transport (*i.e.*, the localisation length or inverse participation ratio) can therefore provide an even more direct proxy of the carrier mobility.^39,40^ These proxies are valid for disordered polymers in which charge conduction occurs primarily along the backbone, but it would be remiss not to also mention the interesting class of organic radical TEMPO-based polymers,^41^ which lack a conjugated backbone and instead exhibit predominantly inter-chain transport between pendant redox centers.^42^ These materials of course require different modelling approaches, but draw from much of the same toolkit.^43,44^

Success in computation of proxies for carrier mobility, and linking them to the structural features of conjugated polymers, is of course heavily influenced by the choices that are made in both the way in which a polymer model is first generated, and the quantum chemical method employed on that model. For the latter, a variety of different techniques have been used. Nelson *et al.* used semiempirical INDO/S calculations to compute the DOS of disordered poly(3-hexylthiophene) (P3HT).^45^ Grimm and co-authors assessed the accuracy of semiempirical methods to study chromophore localisation in poly(*p*-phenylene vinylene).^46^ The groups of Vukmirovic and Troisi both developed specialised tight-binding models for the evaluation of the electronic properties of SCPs^47,48^ and, as a more general approach, the density functional tight-binding method^49^ (DFTB) has also been utilised,^50^ with large contributions here from Elstner and co-workers.^51^ The steady increase in computational power over the years has seen calculations applying density functional theory (DFT) to semiconducting polymer models become the norm, though, with a number of recent examples in the literature.^19,35,52–54^

**Fig. 2:**
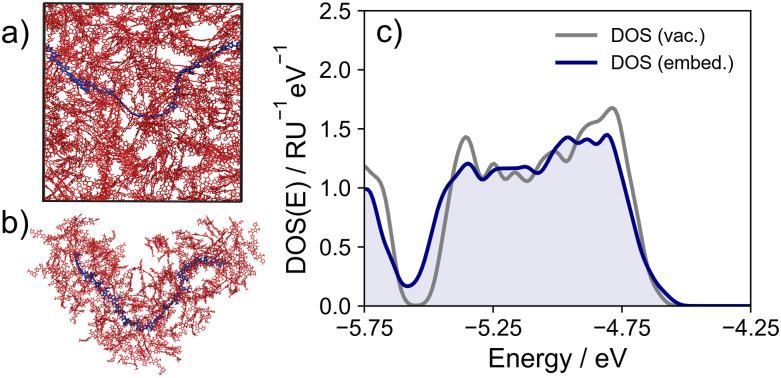
(a) Example of a ‘bulk’ model of indacenodithiophene-co-benzothiadiazole (IDT-BT) – an equilibrated MD simulation box containing tens of chains. Chain conformation is determined (in part) by interactions with other chains. Reproduced from ref. 22. (b) The local environment of a particular polymer chain within the simulation box. The point charges of atoms within this environment can be fed into electrostatic embedding QM/MM calculations of the chain. Reproduced from ref. 22. (c) Valence band of DOS as an average over an ensemble of chain conformations with – (blue shaded line) and without electrostatic effects.

Initial generation of large-scale polymer models has mostly been performed *via* MD simulation, although some studies have diverged from this approach. As an example, Manurung developed a method to rapidly evaluate the electronic structure of amorphous donor–acceptor polymers by (i) building polymer models *via* Boltzmann sampling of torsional angles and their energies from calculations performed only on monomer pairs, and (ii) computing their electronic structure in a similar manner to the tight-binding model introduced in ref. 48.^40^ While successful in quickly obtaining the electronic properties of large numbers of disordered SCPs – essential for any elucidation of concrete design rules – any approach based on procedural generation of chain conformations precludes the inclusion of any effects on the electronic properties arising from interactions between chains, as the microstructure is not explicitly modelled. These effects could come from an additional conformational order/disorder imposed by interactions between chains, or be electrostatic in nature. MD simulation can account for these effects by producing accurate microstructural models from which chain conformations can be sampled, but adds significant computational expense due to long equilibration times. An additional concern of ‘geometry mismatch’^55^ between the classical force-field and QC method used can lead to significant errors in computed electronic properties, although recent advances in accelerated equilibration techniques^22^ and tools for generating QM-parameterised classical force-fields^22,56^ have taken steps to mitigate both of the aforementioned problems. Sampling from atomistic MD models has been used numerous times in conjunction with quantum mechanics/molecular mechanics (QM/MM) methods to account for the effect on conformation of interchain interactions (given an accurate ‘bulk’ polymer model such as that shown in Fig. 2a) and the effect of electrostatic disorder (see Fig. 2b).^19,35,52^ In particular, it has been shown that the impact of electrostatics can vary quite considerably from polymer to polymer and thus cannot be neglected.^22^Fig. 2c shows the DOS computed from MD snapshots for a typical polymer, both with and without the electrostatic interaction.

A number of attempts have been made to extract electronic properties from coarse-grained MD models.^57^ Gemunden *et al.* used a two-stage back-mapping procedure to obtain, from equilibrated coarse-grained models, atomistic morphologies for use as input in electronic structure calculations.^58^ Coarse-graining greatly decreases the computational expense required for simulation and the longer length- and timescales achievable mean it can more easily deal with the effects of mechanical deformation^59^ and processing,^60^ but this advantage is somewhat currently offset by the human effort needed to derive accurate electronic properties from these models. Back-mapping from coarse-grained to atomistic models involves complicated procedures that are not readily automated to ensure that the correct spatial resolution of the atomistic model is retained. Jackson and colleagues have been at the forefront of developing machine learning approaches that bypass the back-mapping procedure in directly mapping the conformationally-dependent electronic structure of molecules to the CG models^61,62^ and, later, in mapping CG representations to all-atom property distributions *via* Gaussian process regression.^63^ Similar approaches have been taken to predict the optical properties of polymers from CG representations.^64^ High-throughput schemes,^65^ which have so far mainly been employed for single polymer chains in vacuum or with implicit solvent,^66,67^ have also incorporated ML approaches for optical and electronic property prediction^68–71^ and for the determination of correlations between monomer-level properties to inform monomer design.^72^

## 3. Phenomenological models of transport

A ‘phenomenological’ model is one that describes an empirical relationship between phenomena, but is typically not derived from first principles. In the case of charge transport in disordered organic semiconductors, this involves describing experimental observations such as the charge carrier mobility with a model parametrised by macroscopic charge transport characteristics. To model transport with such an approach, it is typical to combine some description of the energy distribution of localised states with an evaluation of the transition rates between those states, from which a macroscopic estimate of the mobility can be computed. The Gaussian disorder model^73^ (GDM) assumes that the states which carriers hop between have a Gaussian distribution in energy:1
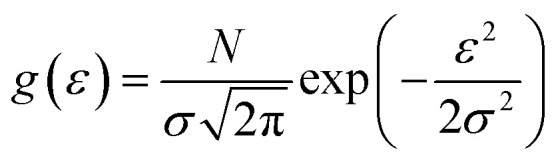
where *σ* is the energy scale of the DOS and *N* is the concentration of sites. The GDM has a number of extensions (*e.g.*, the correlated disorder model^74^), and models which describe the DOS as taking an exponential form have also seen significant use,^75,76^ although the use of a Gaussian DOS is now generally seen as more physically accurate.^25^ Given a distribution of site energies, there are numerous ways to compute pairwise transition rates between states sampled from this distribution. The Miller–Abrahams (MA) rate^77^ between states *i* and *j* describes hopping as a combination of downhill tunnelling and thermal activation:2
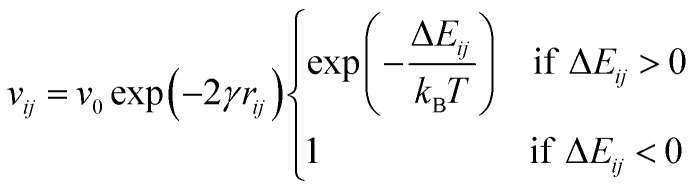
where *v*_0_ is the attempt-to-escape frequency, *γ* the inverse localisation length, *γ*_*ij*_ the distance, and Δ*E*_*ij*_ the energy difference between states. Due to its simplicity, the MA rate is often used in multiscale approaches, particularly to compute transition rates for input in kinetic Monte Carlo schemes (KMC), a tool used to stochastically propagate a system and thus to determine the carrier mobility in various multiscale models.^78–80^ Another common expression is the Marcus rate,^81^ which accounts for the regime of strong electron–phonon coupling:3
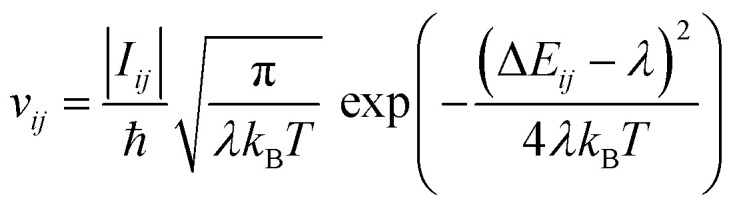
including the reorganisation energy *λ* and the magnitude of the transfer integral *I*_*ij*_. The MA and Marcus rates can be seen as two limiting cases (small- and large electron–phonon coupling) of a more general model.^82^

There are a number of publications that have performed comprehensive surveys of traditional phenomenological approaches^25,83,84^ to the problem of charge transport in disordered organic semiconductors, and we feel there is no urgent need to lengthen this list. Instead, the remainder of this section will discuss approaches that have sought to connect the (often neglected) directionality of charge transport in polymers with the charge transport characteristics, within the (at least partial) framework of a phenomenological model.

**Fig. 3:**
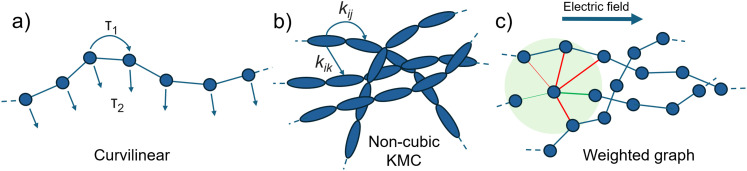
Different phenomenological approaches proposed to include the anisotropic nature of charge transport in conjugated polymers. (a) The ‘curvilinear’ approach taken by ref. 32 assumes 1D transport along a chain interrupted by hopping to a separate chain according to set hopping timescales *τ*_1_ and *τ*_2_. (b) Non-cubic lattice KMC approaches as described in ref. 89 allow the spatial disorder of sites to be included in the charge transport simulation. (c) The directed weighted graph approach of ref. 90, which represents sites as nodes and weights the transfer probabilities between them (edges) according to intra- (green) or inter-chain (red) interactions and the direction relative to the electric field.

Most conventional models of charge transport in disordered organic materials consider transport as isotropic, which can be an acceptable approximation for small molecules but fails to delineate between the competing effects of intra- and inter-chain hopping in polymers. Pearson *et al.* published one of the first investigations into this anisotropy,^85^ using an analytical relationship between the conductivity of a polymer, the time taken for a charge carrier to completely explore a chain *τ*_i_, the mean lifetime for a carrier on a chain *τ*_c_, and the molecular weight. In the case that *τ*_i_ > *τ*_c_ (*i.e.*, the high molecular weight regime), the authors found an independence of conductivity and molecular weight and a predictably linear dependence in the inverse case, when *τ*_c_ > *τ*_i_ and the polymer is limited by the inter-chain hopping rate. Later, Carbone *et al.* presented an analytical relation between charge diffusion, intra- and inter-chain hopping timescales, and inter-site distance in a coarse-grained model^32^ (Fig. 3a). Modelling the case of much faster intra-chain transport within three distinct regimes of flexibility; a rod-like chain, worm-like chain, and Gaussian chain, the authors found that diffusion predictably increases with polymer rigidity, but also that the timescale for inter-chain hopping becomes increasingly irrelevant with rigidity and that the inter-chain transport is never the rate-determining step unless the polymer chain length is exceedingly short. Huang and Rumyantsev recently built on this work,^86^ grouping transport into ‘captive’, ‘semi-free’, and ‘free’ regimes according to the hopping time ratios, degree of polymerisation, and chain stiffness.

Other publications that have studied morphology-dependent transport phenomenologically have done so *via* mesoscale charge transport simulations performed on statistically generated topologies. Mendels and Tessler generated three-dimensional lattices of polymer chain sites with energies drawn from the GDM framework,^87^ performing KMC simulations on these realisations. Mollinger *et al.* studied percolation behaviour between crystallite phases arranged on a two-dimensional triangular lattice,^88^ with chains in the amorphous phase described by the wormlike-chain model. Transport was evaluated using KMC with fixed mobilities for the crystallites and Marcus rates for intra- and inter-chain hopping in the amorphous phases. More recently, Balhorn *et al.* linked experimental microstructure directly to mobility by using transmission electron microscopy (TEM) images to inform kinetic structural generation and charge transport simulations.^17^ The timescale separation between intra- and inter-chain transport can also be achieved using KMC simulations in non-cubic lattices,^89^ as shown in Fig. 3b, giving more flexibility at the cost of less analytically transparent results.

Several studies have used graph theoretical approaches to link the spatial and energetic distribution of chains with charge transport. Graphs of a polymeric system are constructed *via* input from some representation of the morphology: the sites between which charge transfer can take place are defined as ‘nodes’, and each node has weighted ‘edges’ linking it between other nodes in its neighbourhood. Edge weights represent the electronic coupling between sites and can be defined by various parameters, such as the type of edge (intra- *vs.* inter-chain), the relative orientation of nodes, or the distance between nodes. Noruzi *et al.* constructed directed graphs (a cartoon of which is shown in Fig. 3c) from high-resolution transmission electron microscopy images of polymer microstructure, computing the effective mobility for a graph as the average of the time taken for a charge to hop along the shortest path between two electrode nodes, for many different electrode combinations.^90^ While this approach provides an efficient way to link an experimental image of the microstructure of a polymer with a reduced description of that microstructure through a graph representation, and then with some measure of the efficiency of charge transport through that system, it is difficult to make a connection with the specific chemical features of a polymer. The authors use empirical values for edge-weights, useful for evaluating different microstructures but less so for polymer chemistries. Van *et al.* instead constructed an undirected graph from a CG-MD representation of P3HT.^91^ The authors fine-grained this CG representation in order to compute the electronic couplings between sites, which were then used as inputs for a KMC scheme to compute the mobility. Results from KMC were compared with descriptors of the morphology derived from the computationally inexpensive graph approach, with a number of metrics correlating well.

An issue which is common to the graph-based studies detailed above and many other phenomenological approaches is the absence of chromophore delocalisation within the model: it has been shown through both experiment and theory that carriers can be delocalised over a significant portion of a polymer chain.^92,93^ In the models discussed in Fig. 3, states are localised to specific lengths, limiting the distance a carrier can travel in an individual hopping event and artificially reducing the computed mobility. Some authors have attempted to include the effect of delocalisation in phenomenological approaches. Importantly, employing a phenomenological model, Ghosh *et al.* established a relationship between the optical properties of polymer films and two-dimensional polaron coherence length^94^ – a finding that has seen significant implementation in experimental work.^93,95^ For a thorough account of the relationship between optical spectroscopy and delocalisation in conjugated polymers, the reader is directed to ref. 96. Elsewhere, Liu *et al.* proposed a generalised Einstein relation to fit experimentally measured mobilities for a wide range of OSCs, incorporating a parameter defining the ‘delocalisation degree’ of the material.^97^ Balzer and colleagues developed a delocalised KMC approach (dKMC), mapping the time evolution of a tight-binding model Hamiltonian modelled by the polaron-transformed Redfield equation onto a KMC framework, and tracking the probabilistic trajectories of polaronic states.^98^ The authors found increased mobilities compared to standard KMC, emphasising the importance of modelling delocalisation, but the approach was limited by its computational expense. Derewjanko *et al.*, studying conductivity in highly-doped SCPs, derived an analytical expression for the energy-dependent localisation length from a model Hamiltonian based on the GDM.^99^ They used a modified VRH model to compute transition probabilities and fit corresponding conductivities to experimental conductivity-charge density curves.

The area of phenomenological model-based research into charge transport in SCPs has proved successful in that it has been able to describe very well, in a general sense, the transport behaviour of these materials. These models will no doubt also continue to be central in the development of SCP technologies, with recent exemplar applications including the areas of polymer for thermoelectric applications^100,101^ and in further development of models that link spectroscopic and theoretical approaches.^102,103^

## 4. Charge transport from model Hamiltonian

As the phenomenological approaches detailed above have proved successful in describing SCP performance in a general sense, one assumes the goal is to combine these models with detailed electronic structure calculations of atomistic models and attain stronger links between chemical structure and charge mobility – something that is necessary to include due to the numerous examples of chemical specificity determining polymer electronic properties.^104,105^ Standing in the way of this type of study are the mismatches between (most) phenomenological models and electronic structure calculation. Atomistic models can provide an energy-dependent density of states and orbital localisation characteristic, as well as the spatial configuration of sites. Phenomenological models, as we have seen, usually assume an analytic density of states and constant (energy independent) orbital localisation. This implies a distance dependence of the rate that does not depend on the energy of the states – at odds with results that suggest that higher-energy states are more delocalised.^35^ Computing mobility/hopping rates instead from a purely atomistic representation of polymer chains introduces difficulties in determining initial and final states (for intrachain electron transfer), as well as in evaluating electronic and electron–phonon coupling terms.

**Fig. 4:**
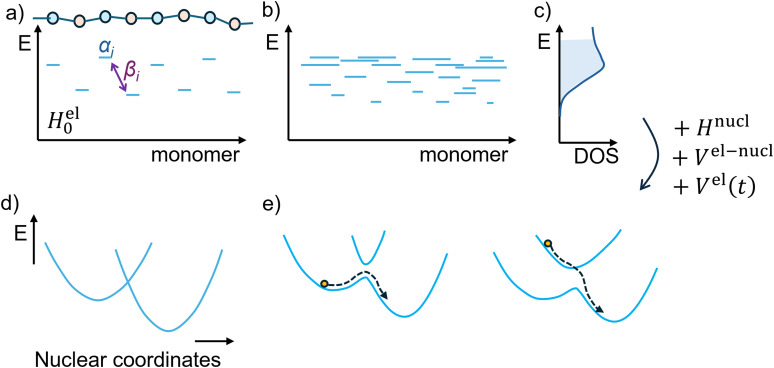
An overview of different modelling aspects for polymer semiconductors. (a) Schematics of a model electronic Hamiltonian (eqn (5)) illustrating disorder in the on-site energies *α*_*i*_ and coupling *β*_*i*_. (b) Diagram of the eigenstates of the electronic Hamiltonian illustrating their variable delocalization (length of horizontal lines) and energy distribution. (c) Density of states computed from such eigenstates, forming the basis of variable range hopping models (eqn (2)). The explicit calculation of the dynamics requires the inclusion of nuclear modes and their coupling with the electronic modes (eqn (4)). One can compute only the charge transfer rate between two states, for example using a diabatic representation as in eqn (3) (d), or explore explicitly the time dependent dynamics (e), for example using surface hopping methods. Solid blue lines represent potential energy surfaces, and dashed lines show the trajectories of charge carriers.

In an attempt to surmount some of the problems arising from these mismatches, a number of studies in recent years have aimed to evaluate the charge transport properties of conjugated polymers through tight-binding model Hamiltonian approaches, building on studies performed for molecular systems.^60^ Many early models focused on the coupling of charge carrier dynamics with the lattice dynamics (often treated classically), neglecting the role of disorder and consequently describing a coherent transport mechanism.^106,107^ As the transport is invariably thermally activated and therefore incoherent, the next generation of models tried to introduce the effect of disorder in the first-order Hamiltonian (*i.e.*, before the electron–nuclear interaction and non-adiabatic coupling terms). These second-generation models can differ considerably in implementation but always consist of a description of the disordered system Hamiltonian (at varying levels of approximation) from certain input parameters (that could be derived from electronic structure calculations). The electronic part of the Hamiltonian can be diagonalised to obtain the wavefunctions and energies of electronic states, thus allowing for easier extraction of parameters to be used in hopping rate expressions and computation of the mobility (see Fig. 4a–c). A general, one-dimensional, coarse-grained model Hamiltonian with explicit change of delocalisation can be written as:^108^

4 *H*^tot^ = *H*^el^_0_ + *H*^nucl^ + *V*^el–nucl^ + *V*^el^(*t*)

*H*^el^_0_ is the electronic Hamiltonian incorporating the static disorder, *H*^nucl^ is the nuclear Hamiltonian, and *V*^el–nucl^ is the electron–phonon coupling responsible of the polaron formation, *V*^el^(*t*) is a residual (thermal) electronic coupling between electronic states which can be seen as dynamic disorder or non-adiabatic coupling between states. A kinetic model, derived computing the charge hopping rate between states localised by the electronic disorder within a Fermi golden rule formalism (Fig. 4d), showed the importance of considering states with a variable degree of localization.^108^ An interesting feature of a model like that of eqn (4) is that, despite the potentially large number of input parameters, the temperature dependence of the mobility is ultimately dependent only on the effective disorder and weighted (by monomer size) strength of the electron–phonon coupling.^39^ This explains the success of the phenomenological approaches discussed in Section 3 and agrees with the rather universal temperature dependence of the mobility seen experimentally.^24^

Several studies have modelled specific benchmark systems, focusing on a range of physical effects. Friday and Jackson used a model Hamiltonian to compute the electronic structure and then mobility of CG-MD models of a conjugated polyelectrolyte in poor- and good solvent conditions,^109^ assigning on-site energies according to the electrostatic potential at each bead. Barford and colleagues, in one of a series of publications,^110^ performed a multi-scale study of transport in PPV in which a Holstein-type Hamiltonian parametrised from electronic structure calculation and conformation generation was used to compute both intra- and intermolecular hopping rates – in turn used to compute the mobility *via* KMC. Poole *et al.*, in a separate study, investigated temperature-dependent charge diffusion in poly(*para*-phenylene) (PPP) by modelling a one-dimensional tight-binding Hamiltonian made time-dependent by Brownian dynamics of the torsional fluctuations^111^ (with the charge propagated by projection of the instantaneous eigenstates from timestep to timestep). In this way, the authors extended previous work by Albu and Yaron which assumed only adiabatic transport,^112^ and the non-adiabatic transport assumed in ref. 108, to model the shift between the two regimes. Berencei *et al.* later employed a very similar Hamiltonian^113^ – this time propagated by Ehrenfest dynamics – owing to potential trivial crossing associated with the ‘projection’ assumption adopted in ref. 111 (although the Ehrenfest approach is not without its own drawbacks – see Section 5).

A number of works have utilised simplified model Hamiltonians to study generalised donor–acceptor (D–A) systems starting with the common tight-binding Hamiltonian:^114^5
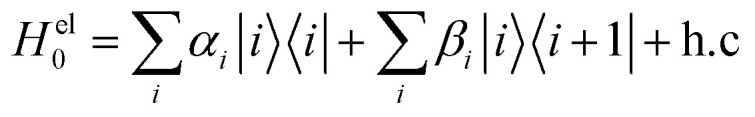
To model a D–A system, *α*_*i*_ (the on-site energy) was set to alternating values for even and odd values of *i*, with *β*_*i*_ distributed around an average value *β* with standard deviation *α*_*β*_ (modelling the coupling between sites and disorder in this coupling). An observation from such a model is that, increasing the energy gap between orbital on donor and acceptors decreases the electronic bandwidth but it is not detrimental to transport (up to a limit). It is indeed the case that successive generations of highest mobility polymers in the past 40 years (PPV, P3HT, PBTTT, IDTBT) tended to have smaller bandwidth as the mobility improved.^108^ Jackson *et al.* adopted the electronic Hamiltonian of eqn (1) to further investigate the properties of D–A copolymers.^115^ Instead of perfectly alternating donor and acceptor sites, a Markov model was used to stochastically generate donor–acceptor sequences in polymer chains. From here, mobility was evaluated using rate expressions for the (i) localised polaron hopping, (ii) delocalised polaron hopping, and (iii) electronically coherent diffusion regimes. The authors found that, in the delocalised polaron hopping regime most relevant to these materials, both alternating D–A and block copolymers exhibited better mobility than randomly generated sequences. An interesting result derived from this approach was that even a minor deviation from an alternating structure eliminated improvements in charge mobility observed for D–A polymers when compared with homopolymers. The impact of polymerisation defects on transport, including the formation of homocouplings^116^ in D–A copolymers and chain branching,^117^ is ultimately unclear and system-dependent^118^ and is not commonly modelled, although the ability of experiment to now quantify defect extent^119^ at the molecular scale offers the challenge of including the correct statistical prevalence of defects into polymer models.

The clear main benefit of model Hamiltonian approaches is that they can be quite easily linked to the microscopic details of a material through atomistic calculations, with microscopic models of charge transport best suited to identifying the limit of validity of the model (*e.g.*, the shift towards coherent transport for strongly ordered phases or the localisation of carriers into a single repeat unit in the case of strong electron–phonon coupling). Model Hamiltonians therefore offer an ideal testbed to validate any model with advanced quantum transport theories,^120,121^ or explore alternative approximated models.^122–124^Fig. 4 also illustrates how such models can be used to offer a synoptic view of the modelling strategies and their connections.

## 5. Fully atomistic models of transport in polymeric systems

Several ambitious efforts have been made to evaluate the charge transport of polymeric systems directly from fully atomistic models. An early approach by Vukmirovic and Wang used a specialised tight-binding model to perform electronic structure calculations on disordered P3HT systems of a few thousand atoms,^125^ calculating electron–phonon coupling constants and transition rates between electronic states. By repeated calculations performed on different MD-produced snapshots of these systems, successively larger models of the polymer were generated, and the authors built a picture of charge transport in the material ranging from lengths on the order of angstroms to hundreds of nanometres.

More recent attempts have tended to follow semiclassical nonadiabatic dynamics schemes, wherein the wavefunction is decomposed into nuclear and electronic parts; the electronic part propagated quantum mechanically and the nuclear part classically (propagation of the combined wavefunction is only available for very small systems and short timescales). Of these semiclassical schemes, simulations based on Ehrenfest dynamics^126^ and fewest-switches potential energy surface hopping (FSSH)^127,128^ have seen the most use in simulations of organic semiconductors – particularly in a range of high-quality studies focused on excitonic processes,^129,130^ with approaches developed in tandem with the charge transport simulations detailed below. Ehrenfest dynamics uses a mean-field approximation wherein the electronic and nuclear wavefunctions interact *via* their respective expectation values, but is limited by its failure to treat trajectory branching/decoherence of electronic states and hence hopping transport.^131^ More appropriate to simulations of disordered semiconducting polymers, the FSSH scheme treats branching by propagating the nuclei on adiabatic potential energy surfaces. Sudden hopping events – the probabilities of which are determined based on transition rates calculated from an auxiliary electron wavefunction that is decomposed into many adiabatic electronic states. The scheme evolves with stochastic hops between these states, as shown schematically in Fig. 4e. There are limitations to this approach: the decay of electronic coherences is incorrectly described,^127^ and ‘crossings’ between potential energy surfaces are not treated properly (which can lead to unphysical long-range transfers).^132^ Semiempirical fragment-orbital based approaches (FOB-SH) have been used to account for these issues.^133^ Here, the charge carrier wavefunction is expanded into a basis of weakly interacting, localised sites which are treated independently. These approaches were found to be internally consistent (in the limit of small couplings between states) and to satisfy detailed balance,^134^ while having the advantage of good scaling with system size, but have the obvious downside of requiring parameterisation. Wang *et al.* introduced a parameter-free crossing-corrected algorithm (CC-FSSH),^135^ which has seen some use in one-dimensional surface hopping simulations of SCPs.^136^ It is worth comparing the radically different timescales accessible to non-adiabatic dynamics (up to approximately 100 ps) and classical (adiabatic) MD (generally exceeding 200 ns). Classical MD can be used to explore large conformational changes of a polymer, including its mechanical properties and potentially its phase behaviour.^31^ Non-adiabatic dynamics simulation normally explores a quasi-static snapshot of the polymer model with better statistics obtainable only by generating an uncorrelated sample of the polymer with classical MD.

Further approximations are often required in these studies: the carrier wavefunction may be propagated within model Hamiltonians^137,138^ – for example with monomers defined by only one electronic state – rather than treating the system fully atomistically. Use of model Hamiltonians to describe the system is complicated by the fact that these models must be initially parameterised from other quantum chemical calculations rather than computing things on the fly, but this can be appropriate for systems where frontier orbitals can be considered localised to specific sites. Heck *et al.* developed a method somewhere in-between full *ab initio* Ehrenfest dynamics and model Hamiltonian approaches to model charge transport in a range of both amorphous and crystalline small-molecule and polymeric semiconductors.^51^ The method differed from conventional approaches in that only the dynamics of the excess charge carrier were propagated (*via* DFTB), such that the systems were divided into small QM regions and much larger MM regions. Fragment-orbital formalism was used with retention of fully atomistic resolution and no parameterisation of the electronic Hamiltonian. Gao *et al.* similarly used DFTB in a semiclassical Ehrenfest approach to model a range of diketopyrrolo-pyrrole polymers at short timescales.^139^ Prodhan *et al.* used CC-FSSH to model temperature-dependent charge mobility and charge delocalisation in P3HT.^136^ This approach was simplified (in order to model large systems) by representing polymer chains one-dimensionally, and reducing the system to a model Hamiltonian composed of monomer-localised states parameterised by first-principles calculations and coarse-grained MD simulations. Nonetheless, the authors found good agreement with experimental data.

Fully atomistic charge transport models have advantages compared to phenomenological models in that they can (i) explicitly model the temporal propagation of the charge carrier with a direct link to the chemical structure of the polymer, and that (ii) they generally make no prior assumptions concerning the transport regime involved. However, there remains the problem that these models are not very scalable to larger numbers of polymers owing to the computational resources required and are usually restricted to short timescales. As for any direct propagation method, the simulation time depends on the phenomena to be observed and, in the case of charge transport, direct propagation of the charge carrier becomes too slow if the charge transport is too slow (*e.g.*, for a highly disordered polymer or with the presence of trap states). The interpretability of the results can also be significantly harder than for simpler electronic structure calculation methods (*i.e.*, those described in Section 2) or model reduction, and complex *post hoc* analysis is often required. Compromises can be made by adopting approximate model Hamiltonians that are propagated in the quantum dynamics, but these require extensive pre-parameterisation – a process which could be difficult to scale for studies on large numbers of polymers.

## 6. Model reduction

Model reduction is the general approach used in science to build simplified lower-dimension models from more detailed descriptions of the systems. In the case of SCPs, the goal is to combine the excellent insight that can be achieved from model Hamiltonians and the requirement for chemically specific parameters that can drive the design of new polymers. The advantage of model reduction with respect to fully atomistic modelling is the ability to study in principle more systems, larger systems or more complex phenomena – a topic discussed in more detail in a recent perspective.^140^ Models with *ca.* 100 000 atoms and hundreds of parameters can often be reduced to effective models with only 2–5 key parameters. The physics of transport can therefore be understood with limited phenomenology, and knowledge of the underlying chemical model is unnecessary. For polymer design however, small structural changes can dramatically affect the mobility, and the availability of a detail model establishes this connection. Early reduced models for semiconducting polymers, like the Su–Schrieffer–Heeger model^141^ or the Pariser–Parr–Pople model,^142^ are still widely used for specialist applications^143,144^ but, by considering explicitly all pi-electrons of the system, they are limited to relatively small repeat units and cannot be easily parametrized for the variety of molecular units seen in modern polymers. On the other hand, polymers of current interest are copolymers containing two or more monomers within the repeat unit resulting in a relatively narrow band near the band gap. For these materials the valence orbital of the polymer can be described as a linear combination of the HOMO orbitals localised on the monomer with the highest HOMO,^65,93^ and it is possible to reduce the dimensionality of the electronic Hamiltonian by considering one orbital per monomer.^40,61^

**Fig. 5:**
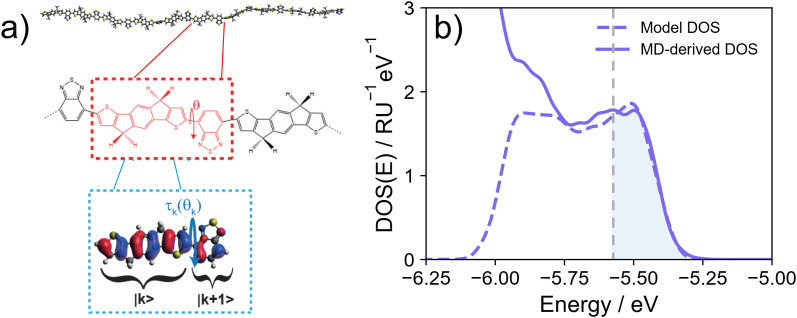
(a) Model reduction approach in which generated chain conformations (IDTBT is shown as a prototypical polymer) are reduced first to the sequence of dihedral angles between fragments. One state (HOMO) per fragment site is considered with the coupling between sites determined by the dihedral angle. Reproduced from ref. 145. (b) An example redrawn from ref. 76 of the result of fitting the valence band of the DOS generated from a model Hamiltonian to the DOS computed from an MD model of a representative p-type polymer. The shaded area represents the part of the DOS to which the model is fitted.

An attempt to build reduced models in a systematic way proposed by Prodhan *et al.* using by DFT-computed torsional potentials and on-site energies mobility^145^ (see schematic in Fig. 5a). The model was built to enable the computation of the charge mobility with an existing model^108^ and, being deployed for 28 polymers, allowed the observation of clear patterns, such as the larger extent of delocalisation in polymers containing fewer repeat units.^146^ With the consideration of a comprehensive set of real polymers, one can also control the individual parameters directly linked to the chemical and structural features of the polymer – a strategy normally unavailable through experiment or *via* phenomenological modelling. By setting all parameters to their ‘optimal’ values, a theoretical limit for the material class can be estimated.^3^ An additional advantage of projecting a complex system on a simplified model is the ability of identifying possible changes in the transport mechanism.^83^

A different approach to model reduction came from Makki *et al.*, who computed the electronic structure of MD-derived chain conformations for a dataset of over 100 polymers.^30^ It is then possible to build a model Hamiltonian similar in form to that of eqn (5) that reproduces the DOS computed from the atomistic model. In practice, one defines a squared residual function *ε*(*E*) = (DOS(*E*)_*α*,*β*,*σ*_*α*_,*σ*_*β*__ − DOS(*E*)_MD_)^2^ measuring the difference between the reduced model and the atomistic DOS. The parameters of the reduced model are those minimising the integral of *ε*(*E*) over an energy range of interest. An example of this fitting procedure is shown in Fig. 5b. By reducing the complex electronic structure of a polymer to the simplest model that adequately describes the system, information that is difficult to obtain from quantum chemical calculation is trivially derived after model reduction. The main advantage of projecting atomistic electronic structure to a model system is the possibility of comparing across systems and establishing interesting correlations. It was found for example that the on-site disorder *σ*_*α*_ – rather than off-site *σ*_*β*_ as previously theorised^35^ – was the largest component of electronic disorder in these polymers. There are still important limitations, however, as the model only considers the electronic Hamiltonian and is insufficient to extract the charge mobility.

Another possible application of reduced models is in the construction of statistical or (machine learned) surrogate models for the mobility. Such statistical models require suitable physical descriptors of the system to be more generalisable, and the parameters of a reduced Hamiltonian are ideal candidates for this role. Alternative approaches to the high-throughput evaluation of polymer electronic properties, such as those using machine learning on coarse-grained representations,^147^ will doubtless also see further development in the coming years. Owing to the complexity of the problem, digital discovery is only slowly entering the domain of polymer science,^148^ but the extension of the burgeoning field of high-throughput polymer simulations^149^ to SCPs is beginning to get underway.^30^

## 7. Conclusion

As shown in this review, the development of models for semiconducting polymers at different scales and resolutions took place in parallel and almost independently. Coarser phenomenological models had great impact in applied physics, model Hamiltonian studies have been critical to elucidate microscopic mechanisms and quantum dynamics, while atomistic simulations have been regularly reported for every key innovation in chemical composition. The respective methodologies have become established, including by improved and generally available software, with robust connections between them that increase the level of confidence in the models. As this is a fairly recent achievement of a large community of scientists with different backgrounds, the various methodologies have not been consolidated yet into workflows for the discovery of new polymers and are still used retrospectively to rationalise existing experimental results and materials. The field still faces key challenges, one being the explicit evaluation of site energies accounting for polarisation effects. The effects of electron–electron and electron–ion interactions on site energies were included in a recent model Hamiltonian approach for the electronic structure of highly doped polymers,^150^ but this is not yet a widespread practice. The strong coupling between ionic and electronic degrees of freedom highlighted in recent computational works^50,151^ has not been translated yet into quantitative models of coupled mass and electron transport. While multiscale models carry the intrinsic risk of introducing inaccuracies at all scales involved, a broad range of experimental methods impose very tight constraints on acceptable microscopic models across their scale. They include X-ray scattering (for ordered regions),^152^ spatially resolved electron diffraction,^17^ NMR,^153^ and STM^119^ (for local structure), charge carrier spectroscopy^154^ (for localisation characteristics of the carrier), photothermal deflection spectroscopy^155^ (for the tail of the density of states), in addition to electrical device characterisation. Increased validation is expected to increase the level of confidence in the theoretical predictions and promote the development of trustworthy models that can explore the chemical space *in silico*, providing predictions testable at the device scale.

## Conflicts of interest

There are no conflicts to declare.

## Data Availability

This work does not contain unpublished data. Data source from figure is given within their caption.
